# Formative research and development of innovative tools for “Better Outcomes in Labour Difficulty” (BOLD): study protocol

**DOI:** 10.1186/s12978-015-0028-5

**Published:** 2015-05-26

**Authors:** Meghan A Bohren, Olufemi T Oladapo, Özge Tunçalp, Melanie Wendland, Joshua P Vogel, Mari Tikkanen, Bukola Fawole, Kidza Mugerwa, João Paulo Souza, Rajiv Bahl, A. Metin Gülmezoglu, WHO BOLD Research Group

**Affiliations:** Department of Reproductive Health and Research including UNDP/UNFPA/UNICEF/WHO/World Bank Special Programme of Research, Development and Research Training in Human Reproduction (HRP), World Health Organization, Avenue Appia 20, 1201 Geneva, Switzerland; Department of Population, Family and Reproductive Health, Johns Hopkins Bloomberg School of Public Health, 615 N. Wolfe St, Baltimore, MD 21205 USA; M4ID - Leveraging new communication technology for development and health, Snellmaninkatu 15, 00170 Helsinki, Finland; Department of Obstetrics and Gynaecology, College of Medicine, University of Ibadan, Ibadan, Nigeria; Department of Obstetrics and Gynaecology, Makerere University, Kampala, Uganda; Department of Social Medicine, Ribeirão Preto Medical School, University of São Paulo, Brazil Av. Bandeirantes, 3900 Ribeirão Preto, SP Brazil; Department of Maternal, Newborn, Child and Adolescent Health, World Health Organization, Avenue Appia 20, 1201 Geneva, Switzerland

**Keywords:** Quality of care, Pregnancy, Childbirth, Obstetric delivery, Obstetric labour complications, Maternal health, Service design, Qualitative

## Abstract

**Background:**

Most complications during labour and childbirth could be averted with timely interventions by skilled healthcare providers. Yet, the quality and outcomes of childbirth care remains suboptimal in many health facilities in low-resource settings. To accelerate the reduction of childbirth-related maternal, fetal and newborn mortality and morbidity, the World Health Organization has initiated the “Better Outcomes in Labour Difficulty” (BOLD) project to address weaknesses in labour care processes and better connect health systems and communities. The project seeks to develop a “Simplified, Effective, Labour Monitoring-to-Action” tool (SELMA) to assist healthcare providers to monitor labour and take decisive actions more efficiently; and by developing an innovative set of service prototypes and/or tools termed “Passport to Safer Birth”, designed with communities and healthcare providers, to promote access to quality care for women during childbirth. This protocol describes the formative research activities to support the development of these tools.

**Methods/Design:**

We will employ qualitative research and service design methodologies in eight health facilities and their catchment communities in Nigeria and Uganda. In the health facilities, focus group discussions (FGD) and in-depth interviews (IDI) will be conducted among different cadres of healthcare providers and facility administrators. In the communities, FGDs and IDIs will be conducted among women who have delivered in a health facility. We will use service design methods to explore women’s journey to access and receive childbirth care in order to innovate and design services around the needs and expectations of women, within the context of the health system.

**Discussion:**

This formative research will serve several roles. First, it will provide an in-depth understanding of healthcare providers and health system issues to be accounted for in the final design and implementation of SELMA. Second, it will help to identify key moments (“touch points”) where women’s experiences of childbirth care are shaped, and where the overall experience of quality care could be improved. The synthesis of findings from the qualitative and service design activities will help identify potential areas for behaviour change related to the provision and experience of childbirth care, and serve as the basis for the development of Passport to Safer Birth.

Please see related articles ‘http://dx.doi.org/10.1186/s12978-015-0027-6’ and ‘http://dx.doi.org/10.1186/s12978-015-0029-4’.

## Background

Labour complications are an important cause of maternal mortality, morbidity and long-term disabilities, particularly in low-resource settings [1,2]. Every year, approximately two million newborn deaths occur globally as a result of intrapartum complications [3]. More than 70% of maternal deaths occur due to direct causes, such as obstructed labour, haemorrhage, uterine rupture, unsafe abortion and sepsis [2]. A substantial proportion of these adverse outcomes occur at home and in the community where women deliver alone or are attended by unskilled attendants [4]. However, many maternal and perinatal deaths occur in health facilities staffed by trained providers [4]. The causes of these deaths are multifactorial, however, late presentation of women with complications to health facilities and delays in implementing life-saving interventions through failure to rapidly recognize and respond to complications are critical contributing factors.

Furthermore, in many health facilities, interventions are used when they are not medically indicated (e.g. labour induction/augmentation, caesarean sections), which can also contribute to otherwise avoidable complications, as well as consuming scarce resources [3,5]. In many low-resource settings, intrapartum care remains suboptimal, particularly labour monitoring, the timely and appropriate administration of effective interventions to prevent and manage complications and the provision of respectful care at birth. Although underlying health systems factors may vary across settings, evidence suggests that staff shortages [6], low morale among healthcare providers [7], knowledge gaps [8], lack of collaboration [9] and poor communication [10] are critical components contributing to suboptimal care [11]. There is general agreement that the identification and appropriate management of women at high risk of labour complications, careful monitoring throughout labour and childbirth, and timely use of effective maternal and newborn interventions (e.g. labour augmentation, assisted vaginal delivery, caesarean section and newborn resuscitation) would avert most of the avoidable intrapartum-related maternal and perinatal deaths [3].

Over the last two decades, strategies to improve labour outcomes during childbirth in health facilities have largely focused on promoting the use of the partograph, which is considered a critical component of labour management. However, its universal implementation has faced many obstacles and it is widely acknowledged that the partograph is poorly and ineffectively used in most low- and middle-income countries [11]. For healthcare providers working in over-crowded and under-resourced health facilities, it is often challenging to process all information relating to labour progress and to take decisive actions during labour management. Furthermore, many health facility and health systems issues related to implementing the decisions made during the course of labour are not captured on the partograph, such as the availability of certain interventions. Therefore, an innovative approach that combines efficient and simple labour monitoring with guidance on the use of appropriate and effective interventions is urgently needed.

Good quality care implies a multidimensional concept that includes, among other factors, appropriate use of effective clinical and non-clinical interventions that are sensitive to women’s values and preferences, strengthened health infrastructure and respectful attitude of health providers, resulting in satisfaction of users and improved health outcomes [12,13]. Efforts to improve the quality of intrapartum care in the facility would therefore only yield desired results by simultaneously addressing community factors that compromise access to skilled intrapartum care. In many low-resource settings, women prefer to deliver in the community due to concerns regarding quality of care in health facilities [12,14-16]. Apart from women’s perceptions of quality of care, the decision-making process around seeking facility-based childbirth care is complex and often influenced by multiple contextual factors. Government and health system policies and public health programmes that encourage women to deliver in health facilities have not been universally successful, due in part to many women’s desires for supportive care, privacy and familiar practices that they experience at home under the care of their birth attendants of choice (e.g.: family members, friends or traditional birth attendants) [12]. Sociocultural and economic influences including the desire for intergenerational continuity of childbirth practices, the role of the male partner and other family members in decision-making, perceived high cost of care and convenience of home birth also play a crucial role in poor utilization of facility-based childbirth care [12,16]. In addition, mistreatment, disrespect and abuse, and negligence by health workers have encouraged dissatisfaction, mistrust, and in many cases complete avoidance of facility-based birth [12,17-19]. As these multilevel effects have far-reaching consequences on obstetric outcomes, a focus on improving the quality of facility care represents the starting point to reverse poor obstetric outcomes in low-income settings.

There is growing recognition that inclusion of the perspectives of those who utilize health services is an important element of the quality improvement process [13-15]. For over a decade, health systems in developed countries have used this approach to improve the quality of health services, and ultimately health outcomes by improving the user experience [20,21]. Yet, the needs, expectations and preferences of women and communities have not been an integral component of designing health services in low- and middle-income countries. Studies have shown that for a health service to deliver high quality care, care improvement should not only focus on views of health managers and staff but also incorporate, value and act upon the experiences and preferences of the users [20-28].

### The WHO Better Outcomes in Labour Difficulty (BOLD) project

The World Health Organization initiated the *“Better Outcomes in Labour Difficulty”* (BOLD) project to address the quality of facility-based childbirth care in low-resource settings [29]. The goal of this project is to accelerate the reduction of childbirth-related maternal, fetal and newborn mortality and morbidity by addressing the critical impediments in the process of labour care and taking advantage of the interactions between the health system and the community. This project seeks to achieve this goal through a two-pronged approach - the development of Simplified, Effective, Labour Monitoring-to-Action tool (SELMA) and the Passport to Safer Birth.

### Simplified, Effective, Labour Monitoring-to-Action tool (SELMA)

Firstly, to address the challenges of labour monitoring (e.g. time constraints and complex monitoring, poor link between monitoring, decision and action, processing of complex patient-level and health system information), the concept of SELMA was developed [30]. SELMA will function as an optimal labour care algorithm as informed by the findings of a cohort study of women delivering in facilities, as well as formative research exploring provider and health system issues related to labour management. The cohort study will collect prospective data on labour management and maternal, fetal and perinatal health outcomes from health facility admission to discharge. From this data, prediction models will be developed to identify women at risk of an adverse intrapartum outcome throughout labour. These models will be used to develop an interactive decision-support tool that would be able to identify the best course of action to avert poor labour outcomes in real time. We envision that the tool will be integrated into a digital interface, for example, as an application to be used on a smartphone, tablet or laptop computer, with the potential to assist health professionals to improve labour management. Additionally, this tool will provide opportunity to optimize task shifting by supporting decision-making of health workers without specialist training (including midwives and non-specialized clinicians), and would thus be useful to health workers working without supervision at lower levels of care to make informed decisions about timely referral to higher level of care. The research protocol for development of SELMA has been published separately.

### Passport to Safer Birth

Secondly, considering that the demand for effective interventions and respectful care can play a strong role in increasing the quality of services provided to women and the coverage of key interventions, the concept of Passport to Safer Birth has been developed. Passport to Safer Birth will be an innovative set of service prototypes and/or tools, co-designed with women, community members and healthcare providers, to promote access to quality care for women and their companions during childbirth. By “service prototypes and tools” we mean two to three tested ideas that improve or enable new interactions between communities and health facilities. For example, a prototype could be a poster, a service guideline, a community meeting or a SMS-based information service. This set of service prototypes and/or tools will take advantage of the benefits of involving women and their communities in designing health services in conjunction with health providers and managers. The tool will be developed using a combination of qualitative research and service design methods, which identifies and prioritizes the needs of the end-users. Service design uses an iterative co-design approach to simultaneously develop solutions and tools to specific problems based on feedback and interactions with the target end-users (in this case, women, their families and facility-based healthcare providers) [25,26]. Given the iterative and dynamic nature of the co-design process, the characteristics or final specification of the service prototypes and/or tool(s) cannot be pre-determined in the early stage of development.

At the minimum, the essential components of Passport to Safer Birth will include negotiated standards of care that are jointly agreed upon by health managers and providers and the communities. By ‘negotiated standards of care’, we mean a level of quality of care that is acceptable and achievable within a specific health facility based on consensus between facility administrators, healthcare providers, women and communities. This process aims to create an atmosphere of collaboration between communities and health professionals, motivate health professionals to subscribe to these ‘negotiated standards’ and at the same time increase demand for these services by the communities. Passport to Safer Birth will essentially function as a support tool for pregnant women and their companions to access quality care and serve as a motivator for healthcare providers. For example, Passport to Safer Birth could take the form of a sim card that provided pregnancy information, antenatal care reminders and risk of labour signs for the woman, and include electronic health records of the woman for the healthcare providers. This solution would be low-cost, fits all phones and potentially connected mobile payment solutions. Table 1 presents the details of the concept and development process of the negotiated standards.

**Table 1:** **Conceptualising and developing the negotiated standards of intrapartum care**

**The negotiated standards of intrapartum care**	A critical aspect of BOLD formative research is the identification of intrapartum practices that are evidence-based, feasible to deliver by the health system and yet align with the values and preferences of pregnant women and their families. These practices, termed the ‘negotiated standards of care’, are intended to underscore the importance of providing humane and respectful care while maintaining high ethical and safety standards in clinical practice. Within this context, the ‘negotiated standards of care’ implies a level of quality of care that is acceptable and achievable within the health facilities based on consensus between health managers and community groups. The development of these standards and the potential benefits when implemented in practice are entrenched in the concept of service co-design by both health service providers and users. The process will create an atmosphere of collaboration between communities and health professionals, motivate health professionals to subscribe to these ‘negotiated standards’ and at the same time increase demand for these services by the communities.
**Development process**	The development of the negotiated standards will occur after the completion of the formative research phase of the BOLD project. The starting points will be the compilation by WHO of existing internationally recognized evidence-based clinical principles and practices for intrapartum care. These principles and practices will be drawn from existing WHO and other international guidelines (e.g. NICE guidelines, midwifery care standards by the International Confederation of Midwives) that are related to the management of normal labour, with due consideration for local contexts. Values, preferences and expectations of potential health service users as they relate to intrapartum care practices will be extracted from the qualitative studies involving health managers, health providers and the community. Similarly, intrapartum clinical practices that are practicable and achievable within the limits of available health resources will be explored through qualitative studies involving health managers and providers of the selected institutions. WHO will process the information from qualitative studies against the background of established evidence-based standards to develop a final list of intrapartum practices or clinical policies where the negotiation between the health managers and providers and community members would start. WHO will mediate the discussion between the health managers, selected health providers, and representatives of the catchment communities to finalise the standards, provide guidance on implementation and encourage providers and users to subscribe to it. A common agreement on what is scientific, feasible and user-centred between health system and community members will lead to service improvement and ultimately better birth outcomes. The adherence to the standards will be assessed in the intervention phase of the project when SELMA and PSB are implemented to improve health outcomes. However, the implementation phase is not part of the current two-year tool development and validation

**Figure 1:**
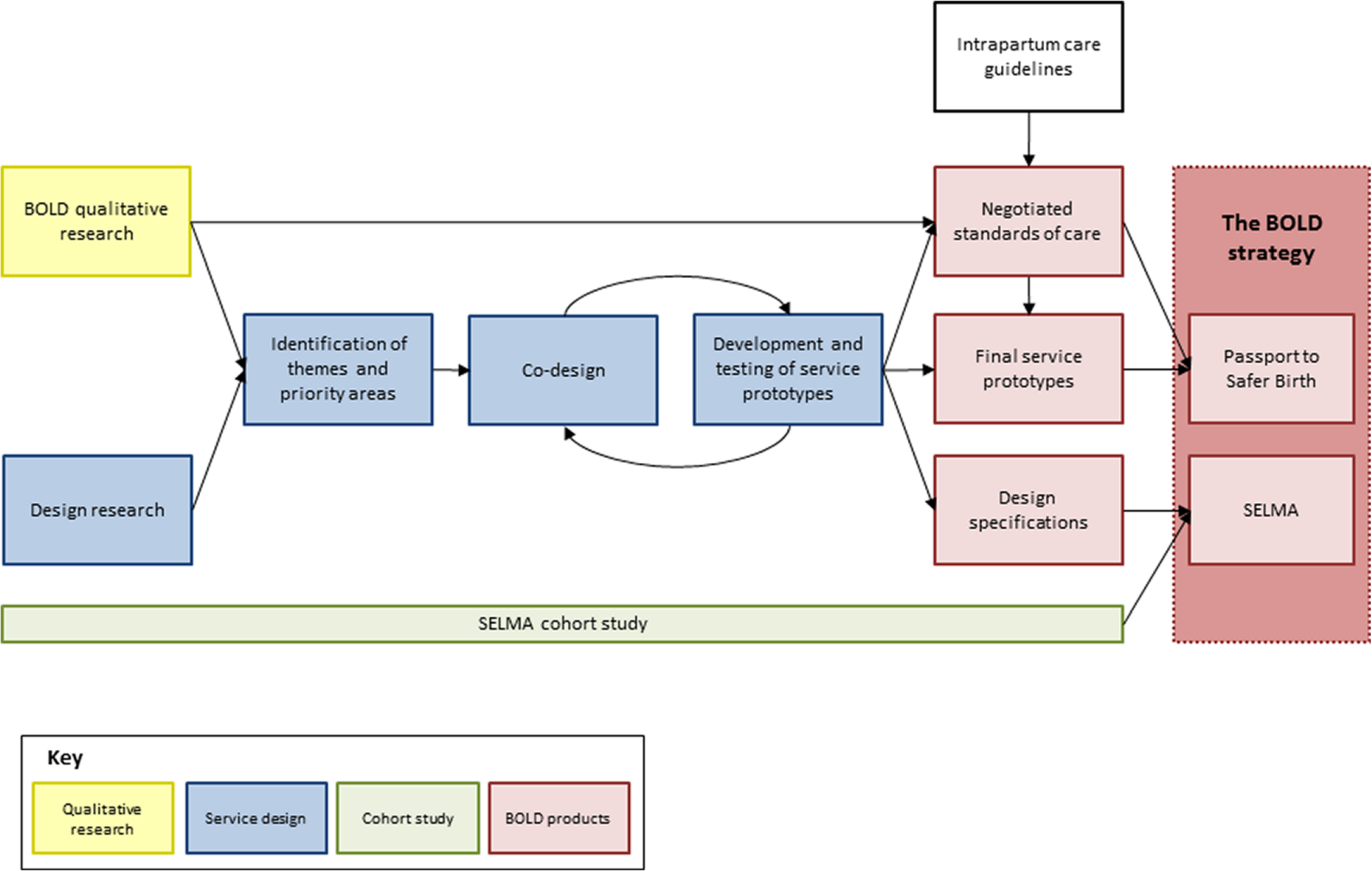
BOLD workflow and analysis plan. (Footnote: Passport to Safer Birth refers to a set of services prototypes or tools. SELMA refers to an electronic labour monitoring-to-action tool).

By increasing the coverage of effective interventions, timely decision-making during labour care and promoting collaboration between health facilities and communities, we aim to improve the quality of care women receive, their satisfaction with care, and health outcomes. The tools developed from this project (SELMA and Passport to Safer Birth) will be integrated into a larger quality improvement approach (the BOLD strategy), which will then be tested in an intervention research study during the second phase of the project. This protocol describes the formative research activities required for the development of these tools. Figure 1 presents the BOLD workflow and analysis plan to demonstrate how findings from the qualitative research, service design and cohort study activities will be integrated into the development of SELMA and the Passport to Safer Birth (the BOLD Strategy).

### Study objectives

The main objective of this component of the BOLD project is to conduct formative research to support the development of innovative tools to improve the ability of health care providers to manage labour and to increase demand for respectful quality care at the time of birth. The specific objectives include:

1. To assess healthcare providers and health systems barriers and enablers to provision of high quality monitoring and delivery of timely, safe and effective interventions during labour and childbirth;
2. To explore the needs and expectations of women and health care providers related to improving the quality of intrapartum care, including birth experiences and outcomes; and
3. To explore and understand the experiences and preferences of community members, women and healthcare providers towards the development of a set of service prototypes and/or tools to improve facility-based labour and childbirth care.

Objectives 1 and 2 are designed to contribute to the development of both SELMA and Passport to Safer Birth, respectively while objective 3 will mainly inform the development of Passport to Safer Birth. For the purpose of this study, “high quality monitoring” (in objective 1) refers to the provision of essential elements of intrapartum monitoring according to international standards which are required to signify the best course of action to prevent adverse outcomes during labour management. At the minimum, these include regular assessment and charting of all components of labour progress on the partograph, including fetal heart rate assessment with the Pinard stethoscope or a hand-held Doppler, assessment of uterine contractions by abdominal palpation (or external tocography), assessment of cervical dilatation and descent of the presenting part of the baby by vaginal examination and abdominal palpation, respectively (every four hours unless otherwise indicated), and monitoring of maternal vital signs, fluid intake and output [31].

## Methods

### General outline

In order to achieve the first and second objectives of the formative research, we will employ qualitative research methodologies among healthcare providers in eight selected health facilities, and among women and community members in the facility catchment areas in Nigeria and Uganda. First, focus group discussions (FGDs) and in-depth interviews (IDIs) will be conducted among different cadres of healthcare providers, as well as IDIs with health facility administrators. These FGDs and IDIs will explore healthcare providers’ and administrators’ expectations and needs relating to provision of quality intrapartum care, as well as barriers and enablers to the provision of high quality care in labour monitoring-to-action. Furthermore, FGDs and IDIs will be conducted among women of reproductive age (15–49 years) who have delivered in any health facility in the previous 12 months to explore their expectations and needs to improve their care experiences during childbirth.

**Figure 2:**
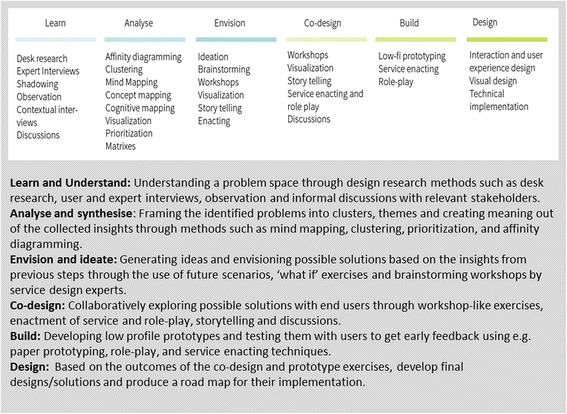
An example of a service design process.

To meet the third objective, we will use a service design approach to provide design specifications for the development of SELMA and Passport to Safer Birth. ‘Service design’ is a multidisciplinary approach to design new or improve existing services around the needs of service users and providers with the aim to make them more useful, desirable, effective and efficient [20,21]. Service design applies user-centred methods to innovate and shape services organised around people’s needs and desires, with consideration of the constraints and possibilities of service providers and environments. Using a service design approach in health care has demonstrated benefits related to improving the creative process, the service provided, project management, and organizational culture [20-28]. The use of creative methods to identify and design new service opportunities in an iterative processes of testing and refining facilitates the development of solutions that enable lasting relationships between service providers and recipients. Although some of its methods are similar to qualitative research methods (such as interviews and small group discussions), design research differs from qualitative research in that it is less formal and uses visual tools of documentation as an outcome to inform and inspire the design team. Design research aims to map a holistic picture of a social phenomena or service environment and outline the interdependences, such as stakeholders, service interactions, perceptions and emotions. Figure 2 describes an example of the service design process.

Within the context of the BOLD project, we will explore healthcare providers’ interactions with the health system and consider available resources, constraints and the context in which they operate. We will also explore the context of pregnant women and their experiences, values and preferences relating to facility-based childbirth. For the purposes of this protocol, the qualitative research design and procedures related to the first and second study objectives will first be presented, followed by the service design approach and procedures related to the third study objective.

### Procedures: qualitative research

#### Study sites

This study will be conducted in eight health facilities and communities within each facility catchment area, in Nigeria and Uganda (four health facilities from each country). These health facilities have been identified for the BOLD project in collaboration with the country principal investigators with consideration of the following inclusion criteria:

- A minimum of 1,000 births per year;
- The major health care facility in its area or district (e.g.: not a primary health care unit);
- Stable access to caesarean section, augmentation of labour, operative vaginal delivery and good intrapartum care practices (e.g. intermittent fetal monitoring at the minimum)

#### Study participants

Three groups of participants have been identified for this part of the study: [1] facility administrators; [2] healthcare providers; and [3] women who have given birth in any health facility in the previous twelve months. From each of the selected facilities, facility administrators, such as the head of the obstetrics and gynaecology department or the head of the hospital, will be invited to participate in IDIs. Healthcare providers from the selected facilities, including midwives, medical officers and obstetricians, will be invited to participate in FGDs and IDIs. FGDs and IDIs will not be conducted with the same healthcare providers to avoid redundancy among participants. If the number of healthcare providers is insufficient to constitute the pre-determined focus group in any facility, only IDIs will be performed among such providers.

Two health facilities in each country will be sampled and women who reside in the catchment areas of these selected facilities will be invited to participate in the study. This process will be facilitated by the country and local study teams. FGDs will be conducted with women of reproductive age (15–49 years) who have delivered in any health facility in the previous 12 months to explore normative expectations, needs, and opportunities to improve their care experiences during childbirth. Additionally, IDIs will also be conducted with women of reproductive age (15–49 years) who have recently delivered in any health facility to explore their personal expectations, needs and opportunities to improve their care experiences during childbirth.

#### Participant recruitment

The country principal investigators, social scientists and local research teams will facilitate contact with women in the communities within the selected facility catchment areas, as well as the healthcare providers and facility administrators from each facility. Each individual will be invited to participate and if they agree, will be asked to provide consent. All FGDs and IDIs will take place in a private setting and will be audio recorded. FGDs and IDIs are anticipated to last approximately 60 to 90 minutes and will be conducted by trained qualitative researchers from the country teams. For the FGDs and IDIs conducted with women who recently delivered, the moderators will be female.

#### Sampling

Once the facilities are selected, the catchment area for each facility will be defined for sampling purposes. Purposive sampling will be used to achieve a stratified sample without random selection. This method uses pre-specified parameters to stratify the sample [32]. The sampling grid (Table 2) outlines the proposed stratification for women, healthcare providers and administrators. In each study facility, healthcare providers will be sampled based on their cadre, such as nurse/midwives or doctors/specialists. In each facility, facility administrators will be selected for IDIs. We expect the type or designation of facility administrators to vary by facility, but at the minimum would include the medical administrative head of the facility and the head of the obstetrics and gynaecology department.

**Table 2:** **Sampling grid to be used in each country (Nigeria and Uganda) for qualitative research to meet objectives 1 and 2**

**Participant type**	**IDIs**	**FGDs**
Category 1: Facility administrators (for 4 facilities per country)	1 to 2	-
Category 2:Facility-based healthcare providers (for 4 facilities in each country)		
Midwives	3 to 5	1 to 2
Doctors	3 to 5	1 to 2
Category 3: Women of reproductive age (15-49) who have delivered in any health facility in the previous 12 months (for 4 facilities catchment areas in each country)	10 to 15	2 to 3
**Total (across 4 study facilities per country)**	**68 to 108**	**16 to 28**

In each country, two facilities will be selected, and women will be sampled for IDIs and FGDs from within their facility catchment areas by the lead social scientist in each country. Women who recently delivered in any health facility will be identified in collaboration with community health workers using community mobilization mechanisms. Women who delivered outside of a health facility will be excluded from this sample, because this project seeks to explore women’s experiences of care provided at the facility-level, rather than barriers and facilitators to facility-based childbirth care.

#### Study instruments

All of the instruments will use the format of semi-structured discussion guides and are available upon request.

IDI discussion guides for facility administrators will include the following domains:

A. Explore the meaning of quality intrapartum care in your work environment.
B. Barriers and facilitators to the provision of quality intrapartum care in your work environment, focusing on labour labour monitoring-to-action.
C. Potential changes to enhance the provision of quality intrapartum care in your work environment, across women, community, provider, facility and health system levels.
D. Perceived expectations and needs of women seeking facility-based intrapartum care, focusing on ideal and exiting communication channels.

FGD and IDI guides for healthcare providers will include the following:

A. Explore the meaning of quality intrapartum care in your work environment.
B. Expectations and needs to provide quality intrapartum care, including labour monitoring and timely interventions.
C. Barriers and enablers to the provision of quality intrapartum care in your work environment, focusing on labour monitoring-to-action.
D. Potential changes to enhance the provision of quality intrapartum care in your work environment, across women, community, provider, facility and health system levels.
E. Perceived expectations and needs of women seeking facility-based intrapartum care, focusing on ideal and existing communication channels.

FGD and IDI guides for women will include the following domains:

A. Perceptions of care provided at the facility and decision-making to seek care at the facility.
B. Knowledge of labour and childbirth practices.
C. Explore the meaning of good quality of care during childbirth in health facilities.
D. Perceived expectations and needs while seeking facility-based intrapartum care, focusing on ideal and existing communication channels.
E. Potential changes to enhance the provision of quality intrapartum care in the health facilities, across women, community, provider, facility and health system levels.
F. Perceived expectations and needs of providers during facility-based childbirth, focusing on ideal and existing communication channels

#### Data quality assurance

Prior to data collection, a one-week training session will be conducted in Nigeria and Uganda for all research teams, including country PIs, social scientists, data collectors, research assistants, transcribers and translators. The training session will include objectives of the study, data collection procedures, practice sessions with the tools, pilot-testing in a health facility and community, as well as highlighting ethical considerations. The WHO qualitative research team with support from the social science team leads of each country will train the country qualitative research teams, which will include social scientists, focus group moderators and interviewers from University of Ibadan, Nigeria and Makerere University, Uganda. The lead social scientist from each country will ensure that experienced moderators and interviewers are invited to participate in the study. A manual of operation (moderator’s guide) will be developed by the WHO team with inputs from country collaborators to standardize the quality of data collection across both countries.

During the data collection period, the transcription and translation will occur in parallel to data collection and will be shared on an on-going basis with the study team to ensure the quality of the data. Country PIs will be in constant communication with the interviewers in the field in order to respond to any issues that arise during data collection. Transcripts will be reviewed throughout the data collection process to ensure data content and quality. A random sample of six transcripts (three per country) will be back-translated into the local language to ensure translation quality.

#### Data management

All digitally recorded qualitative data (group discussions and interviews) will first be transcribed verbatim in the original language used for collection using a structured transcription format. Verbatim transcription will be performed close to the time of completion of the interviews/discussions to maintain the originality of the discussion without loss of themes. Observations and assessments during interviews will be written up as field notes. The transcripts will be complemented with notes taken during the interviews/group discussions. Data transcription will be performed under the supervision of the designated social scientist who will review it for completeness. The transcripts in local languages will be then be translated into English by an independent translator following the original transcription format. All translated transcripts will undergo another round of consistency checks by country lead social scientist to maintain high data quality. The lead social scientists from both countries will manage the audio and transcribed files, and will transfer them electronically to a WHO project staff in charge of BOLD qualitative study at a regular interval that is mutually agreed upon (e.g. weekly or fortnightly). The WHO project staff will be tasked with data management of the transcripts and audio files. Transcripts will be stored in Atlas.ti computer software and stored on a password-protected computer accessible only to the study team. Transcripts will be de-identified and participants will be identifiable only by a unique identifier code. Participant’s names and personal information will not be recorded.

#### Data analysis plan

Thorough debriefing sessions will be conducted between the lead social scientist and the research assistants on a mutually agreed upon schedule to review field notes, adjust interview guides, and identify potential questions or scenarios of interest or confusion to clarify through member checking in subsequent interviews. We will employ a two-pronged approach for the formal analysis: [1] conduct local analysis workshops with the research assistants in each country; and [2] line-by-line coding to develop a thematic framework and coding scheme. In each country, the local analysis workshop will be facilitated by the lead social scientist and the WHO qualitative research lead to foster a hands-on capacity building activity for research assistants engaged in the project and to share insights from the data collection process to develop better understanding of the local context.

The WHO qualitative research lead, in conjunction with the qualitative research team, will conduct line-by-line open coding on a sample of the translated transcripts to develop the thematic framework. The thematic framework will also be informed by the study objectives to explore the barriers and facilitators to the provision of high quality care in labour monitoring-to-action, and expectations and needs of both healthcare workers and women during intrapartum care. The thematic framework will inform the development of a hierarchical coding scheme, which we will apply systematically to all transcripts using Atlas.ti (version 7.1.7 Scientific Software Development GmbH, Eden Prairie, MN). Text units indexed according to each emergent theme will be further analysed and interpreted by the larger study team.

We will explore common themes that span geographic and cultural differences while identifying important differences across settings that need to be accounted for during the tool development stage. As a hospital-based tool that is anticipated to be globally applicable to settings similar to both Uganda and Nigeria, SELMA development will only be informed by themes that are common to both study settings. However, the Passport to Safer Birth will be designed to respond to local issues and context and its development will be informed by themes that are most relevant and specific to study settings. The process of Passport to Safer Birth development will be carefully documented to allow reproducibility and future scale-up to other settings. Close collaboration between the WHO qualitative research team, the lead social scientists, and the research assistants will ensure quality analysis and interpretation of the data across sites.

### Procedures: service design

#### Service design overview

**Figure 3:**
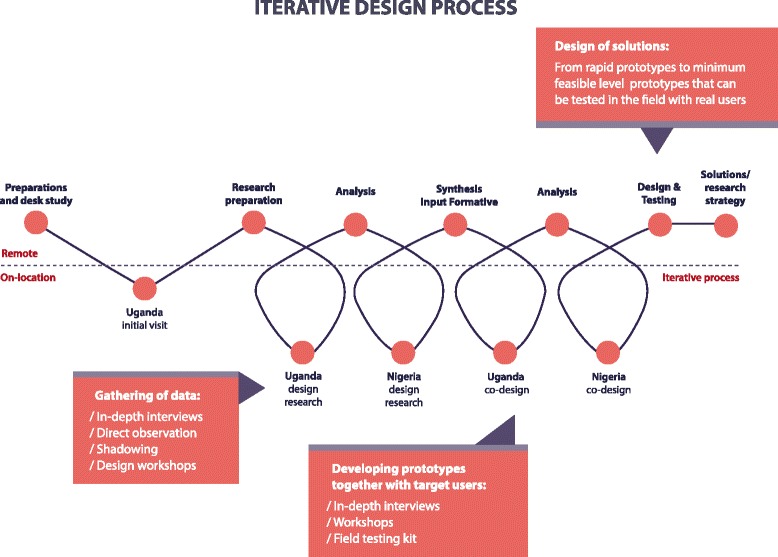
BOLD iterative design process.

The service design approach will be conducted by partner social enterprise organization with technical input from WHO. The service design activities are devised to support the development of the study tools (SELMA and Passport to Safer Birth) to improve access to respectful, quality care during childbirth. Using the service design process, we will identify key moments and places (“touch points”) where women come in contact with maternal health services and where their subjective experience is shaped. These touch points represent the location or time that the desired emotional and sensory connection to health services needs to be established. The preliminary results from the qualitative research discussed in the sections above will also complement and contribute to the outputs from the service design activities. Figure 3 depicts the iterative process used in design research to gather data, develop prototypes with target users and design solutions to be tested with real users.

**Figure 4:**
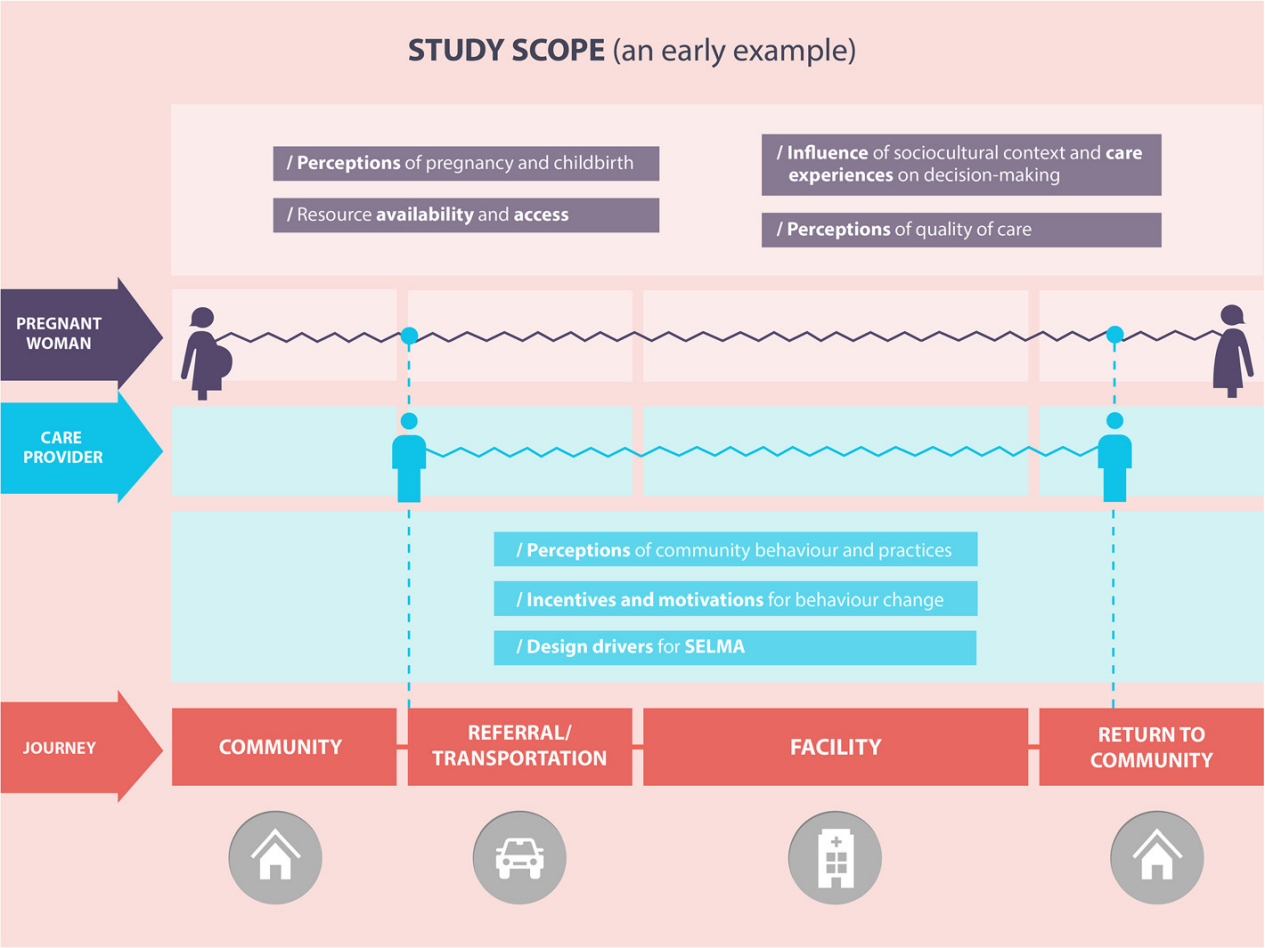
Draft template of user journey map.

In the first phase of the service design process, we will conduct design research in the selected health facilities and facility catchment areas in Nigeria and Uganda to gather insights from both facility staff and women through the use of participatory observations and interviews. Figure 4 depicts the draft template of a user journey map of women from the pre-pregnancy to postpartum period that will guide the design process. This user-journey will be used as a guide during observations and interviews to identify, map and adjust touch points throughout a woman’s journey, as well as understand women’s expectations and needs, stakeholder involvement, barriers, challenges, and opportunities for improvement. As service design employs an iterative approach, insights gathered from the initial round of observations and interviews will influence the structure of the next round of observations and interviews.

#### Participatory observation

We will conduct participatory observations of consenting individuals from the target groups, to explore and engage with their environment, activities, behaviours, interactions with individuals from other target groups, decision-patterns, as well as interplay with physical objects or technology relevant to the BOLD project. The observations will be systematically documented, but will differ slightly in their degree of formality, level of structuring and recording methods (depending on whether the observation is in the community or the health facility). The service design researcher may ask questions or engage participants in conversation, while avoiding interruption to natural routines, activities and interactions. The observations will enable the design team to identify, analyse, prioritize, and visualize existing practices and perceptions around the time of onset of labour, and map the journey of women to and within the health facility.

The observation schedule for facilities will be developed in conjunction with the facility administrator to find a time of day to conduct the observations that respects the workflow within the facilities. For example, if antenatal clinics are only held on weekday mornings, observations for the antenatal clinic will be held on a mutually agreed upon weekday morning. Key points of observation will include the following:

- Shift start and end times and handover period for nurse/midwives and doctors/specialists
- Workflow during antenatal care clinics
- Facility admission and triage processes
- Monitoring of women on the labour wards
- Documentation during labour management (i.e. how labour progress is charted and to what extent partographs are completed)
- Discharge and follow-up process
- Workflow during postnatal care and immunization clinics

Participants and locations for the facility observations will be identified by the facility administrator. As the facility setting is dynamic in nature, it is not possible to specify in advance the number of participants who will be observed. The observations are designed to capture as naturalistic of a setting as possible; therefore, consent will not be asked of every person who would be observed. Observations in the communities will be coordinated with the local partners, which include a local service design team and community focal points. Key points of observation will include community meetings, public social areas in the community (i.e. marketplace or bar), community health worker visits and women’s daily activities in the home and community

Participants and locations for the community observations will be identified by the local partners. Consent will be sought from the community contact person (e.g. community leader) for these observations. As the observations are designed to capture as naturalistic of a setting as possible, consent will not be asked of every person who would be observed in large group gatherings.

When conducting the observations in the facilities and communities, the service design research team will be discreet enough to not disrupt normal activity, yet open enough so that the people under observation do not feel that their privacy is compromised. All relevant people in authority (i.e. facility administrators, heads of wards, community leaders) will be alerted to the presence and purpose of the research team and will provide consent for the observation. If someone under observation queries directly about the role of the research team, an honest, open and polite answer will be provided. If informal discussions occur with those under observation, the research team will emphasize that they are not required to talk to the research team and there will be no repercussions if they chose not to. Confidentiality will be maintained through the period of observations and the identities of those who are observed will be protected and will not be linkable to the data.

Individuals from the following target groups will be selected for the observational sessions: [1] pregnant women (in second and third trimester, including when travelling to the facility); [2] women during labour and childbirth and immediate postpartum (where deemed acceptable by mother/family and considered feasible by health staff); [3] women and their newborn (returning to the community); [4] family members of women; [5] traditional birth attendants; [6] community health workers; [7] hospital reception and admission staff; [8] facility-based nurses, midwives and doctors.

#### Semi-structured interviews

We will conduct semi-structured interviews with facility staff and women within the service environment (i.e. in health facility for both staff and women) or context (i.e. in the community for women), allowing the interviewer to both observe and probe behaviour at the same time. These interviews will help design researchers to gain an understanding of the social and physical environment surrounding the potential service. Consenting individuals from the following target groups will be invited to participate in the interviews: [1] pregnant women (in second and third trimester); [2] women during labour and childbirth and immediate postpartum (where deemed acceptable by mother/family and considered feasible by health staff); [3] women and their newborn (returning to the community); [4] family members of women; [5] traditional birth attendants; [6] community health workers; [7] hospital reception and admission staff; [8] facility-based nurses, midwives and doctors.

The interviews will be conducted based on discussion guides (available upon request), which will be refined following the observations and themes identified in the user journey map. Several visual tools such as illustrations, mood boards and other imagery will be used as discussion help to stimulate thoughts and ideas.

#### Participant recruitment

Eight health facilities and communities within each facility catchment area in Nigeria and Uganda (four health facilities from each country) have been identified for the BOLD project. The service design approach will be implemented in each of the BOLD sites with an additional rural site in each country to provide a different perspective. Healthcare providers from the study facilities will be invited to participate in observation and semi-structured interviews. Women of reproductive age (15–49 years) who are either pregnant or have delivered in a facility in the previous 12 months who reside in the catchment areas of the selected facilities will be invited to participate in observations and semi-structured interviews.

The country principal investigators and local design research partner will facilitate contact with the women in the communities within the selected facility catchment areas as well as the healthcare providers and recently delivered women in the selected facilities. Each individual will be invited to participate, and if they agree, will be asked to provide consent. The interviews will be audio recorded. Interviews are anticipated to last approximately 60 minutes and will be conducted by trained local research partners and design researchers. The discussion moderator for the interviews and observations conducted with women in the community or facility will be female. Observations are anticipated to last at least 60–90 minutes depending on the work context, key points of observation and availability for observation. Purposive sampling will be used to achieve a stratified sample without random selection.

The objective of the Passport to Safer Birth is to create a better linkage between communities and facilities and to stimulate the demand for respectful, humane, and quality intrapartum care. Since a woman’s decisions regarding care-seeking during pregnancy and childbirth is often influenced by her significant others, we will also include other stakeholders in the interviews, including husbands/male partners, in-laws, heads of households and other close family members. These participants will be recruited through the women who participate in the interviews, and the interviewer will ask the woman if their husbands/partners and/or other family members will be available for participation in a separate interview.

#### Service design research instruments

The observational domains for service design will include the following:

In the community:

- Living environment/community environment
- Structure of and influence among the family
- Relations and contact points with family, peers, other influencer groups
- Daily routines of women
- Touch points and interaction with information/education (books, newspaper, radio, TV, mobile, word of mouth, leaflets etc.)
- Touch points and interaction with technology specifically
- Access to and communication modes for transportation
- Touch points and interaction to skilled and non-skilled health care providers

In the health facility:

- Daily routines of healthcare staff
- Structure of the organization at facility
- Workflow with pregnant women and women in labour, childbirth, and postpartum
- Touch points and interactions with technology in facility and links to community
- Communication methods within the facility
- Access to information, learning, education

The research team will follow the above guiding frame during the observations but departures from the plan are anticipated and allowed in response to unexpected events during observation. Documentation of the observations will be through note taking, sketches, and occasionally through photographs, audio recording/or video footage (with consent and when deemed appropriate and not intrusive to the staff and woman). There will be no visual documentation (e.g. photograph or video footage recording) during sensitive medical or personal situations (e.g. breast, abdominal or pelvic examinations, surgical operation, emergencies, experience of labour pain, birth or bereavement). The most important events to observe are preparatory tasks or documentation executed by hospital staff during care provision, activities at critical locations in the health facility (e.g. waiting room/hall, entrance, admission) and medical equipment and materials. Care will be taken by the design team to limit the intrusion into the healthcare setting and to maintain women’s privacy and confidentiality. The design researchers will also carry out a review sessions after each observation day together with the local partners in order to review, clarify, summarize and document the days gathered information. This will then be used, together with the detailed recordings, as the basis for the analysis phase.

Interview guides for women and community members will include the following domains, which will be refined following the observations and themes identified in the user journey map.

A. Traditional influences and beliefs regarding pregnancy and childbirth
B. Health seeking behaviours and touch-points during the antenatal, intrapartum and postnatal periods (including journey mapping from home to the facility)
C. Perceptions and experiences of facility-based intrapartum care
D. Role of and access to communications, technology and media

Interview guides for health workers will include the following domains, which will be refined following the observations and themes identified in the user journey map:

A. Facility-level processes, routines and protocols across the intrapartum care spectrum
B. Healthcare delivery behaviours, experiences and touch-points during the antenatal, intrapartum and postnatal periods
C. Managerial and leadership practices
D. Facilitators and barriers to identification of signs of labour risk
E. Role of and access to communications, technology and media

#### Data quality assurance

Prior to data collection, the service design partners in collaboration with the principal investigators will hold a BOLD service design kick-off meeting with key stakeholders, including study coordinators from each health facility, community health workers from the study catchment communities and local research partners. The meeting will launch the service design activities and provide an overview of objectives, the concept of service design, data collection and analysis procedures and ethical considerations. The service design team will train the local research and design partners on the study tools and procedures. During the data collection period, the transcription will occur in parallel to data collection and will be shared on an on-going basis with the study team. The service design team, local research partners and principal investigators will be in constant communication to respond to any issues that arise during data collection.

#### Data management

The above described service design activities will be coordinated by M4ID, a not-for-profit social enterprise leveraging communication and technology for health and development based in Finland. M4ID will use the findings from the primary qualitative research to inform the service design process, and is responsible for leading the participatory service design component (in conjunction with in-country service design partners), developing the prototypes for Passport to Safer Birth, community testing and refining the prototypes, and a final documentation of a strategic roadmap for the production and implementation of Passport to Safer Birth in future work.

All digitally recorded interviews will transcribed verbatim into English by local research partners. Observations, assessments and preliminary design ideas will be written and drawn as field notes by M4ID service designers and will complement the transcripts. Transcripts will be stored on a password-protected computer accessible only to the study team. Transcripts will be de-identified and participants will be identifiable only by a unique identifier code. Participant’s names and personal information will not be recorded.

#### Data analysis plan

Findings from the service design insights will be organized and analysed using a service design approach, including: [33]:

A. Affinity diagramming: a process used to externalize and meaningfully cluster observations and insights, keeping design teams grounded in data as they design.
B. Visualized mapping: pictorially demonstrating the journey or decision/activity flow for each of the target groups.
C. Personas: consolidate archetypal descriptions of user behaviour patterns into representative profiles, to humanize design-focus, test scenarios and aid design communication.
D. Mapping methods: representation of different networks of ideas and associations in a visual manner, including:
  - Concept mapping: a visual framework that allows designers to absorb new concepts into an existing understanding of a domain so that new meaning can be made.
  - Mind mapping: a method of visually organizing a problem space in order to better understand it, when a topic or a problem has many moving parts.
  - Cognitive mapping: a visualization of how people make sense of a particular problem space, and is most effective when used to structure complex problems and to inform decision-making.
E. Journey mapping: mapping a service users journey throughout each touch point and identify needs, experiences and perceptions that are shaped in each touch point. With the help of a journey, new service opportunities can be identified. The user journey map is both a tool to document design research findings as well as a tool to ideate and map new solutions.

A summary analysis utilizing the above analytic process will be used to develop a prioritized design guideline, which will inform concept ideation, solution design (SELMA design specifications), and the development of prototypes (Passport to Safer Birth).

### Project management

The qualitative research and service design components will be managed by WHO BOLD study coordinating unit, at the WHO Department of Reproductive Health and Research, Geneva, Switzerland. In Nigeria and Uganda, the country principal investigators will establish research teams that will implement the research and design activities. The qualitative research component will be executed by local social science teams in Nigeria and Uganda. The service design component will be executed by M4ID, a service design organization from Finland, in collaboration with local innovation and research partners. The study coordinating unit in Geneva will conduct site visits before and during the implementation of the study to contribute to study site selection, training workshops and assessment of adherence to study protocols. Training of country research teams will take place at convenient sites in both countries. There will be continuous communication between country research teams and study coordinating unit at the WHO. Regular contacts will be made and statutory teleconferences will be arranged to ensure that the timeline are followed and problems resolved without delay.

### Ethical considerations

#### Study population, recruitment strategy and informed consent process

This study will employ broad participation criteria to be as inclusive as possible of all cadres of healthcare providers and women with different life situations (including religion orientation, socioeconomic status, ethnicity, age). Therefore specific sub-groups of healthcare providers or women are not disadvantaged through being unable to participate in the study. Potential participants in the hospital and the community will be identified and invited by trained research staff who are familiar with the facility and the community. All potential participants will receive information about the study in their language of choice, conforming to ethical requirements for research involving human subjects. The language will be easy to understand and free of technical jargons. Participants will be given sufficient time to reflect on the information and ask questions. Those who consent to participate in the study will be requested to sign an informed consent form, and it will be made clear that they are free to withdraw from the study at any stage without risk of any negative consequences. For illiterate women, an impartial witness will be present during the entire informed consent reading and discussion. Both the witness and the individual discussing the consent will sign and date the consent form. The contact details of the local investigators, including telephone numbers, will be made available to the participants should they require further information and assistance.

Other safeguards will include the use of unique participant numbers on all data collection forms, and ensuring that interviewers and data collectors are not current or previous employees of the study facility.

#### Perceived risks and benefits of the study, both at the individual and community levels

It is possible that women who participate in the semi-structured interviews may become upset if they have experienced a traumatic birth experience and the interview revives their feeling of distress. However, most questions on the interview guide will explore women’s expectations and needs during the intrapartum period rather than exploring traumatic birth experiences. Interviewers will be trained on how to support any woman who becomes upset during the interview, including how to initiate and follow up referral to appropriate section of the hospital where the woman could receive psychological support.

Participants will not experience any direct and/or immediate benefits for participating in the study. However, the study will be gathering information to inform the development of tools that have the potential to improve the quality of labour management in the future. Study participants and other women using or intending to use facilities for childbirth will benefit from the increased scientific knowledge on this topic, which will ultimately promote women-centred care of high quality in the facilities.

#### Safeguards to protect any recognized vulnerability of the study participants

Vulnerable or potentially vulnerable sub-populations (such as unmarried women, adolescents, women of different ethnicities, migrant women and women who are HIV positive) may participate in this study. We consider it important to ensure that the selection of participants did not discriminate against any group, as women in this category may be at greater risk of receiving poor quality care in the facility. If such women are included, they will be protected by the universal standards of confidentiality and privacy that apply to all participants. However, all women, including these vulnerable groups, will be free to refuse to participate, both confidentially and without prejudice.

#### Reimbursement or compensation to study participants

All participants in both the qualitative research and service design research activities will receive a small reimbursement to cover their transportation to the venue of the interview. The value of this payment will be determined in consultation with the country principal investigators, to ensure that it does not constitute an inducement.

#### Responsiveness of the project to community needs and priorities

Quality of care during childbirth has been identified as an important issue in low-income settings, and even as a human rights issue. The findings of this study will inform the development of innovative tools to improve how intrapartum care is delivered by health providers and the standard of care experienced by women delivering in the health facilities.

#### Deception

There will be no form of deception in this study.

### Ethics approval

The WHO HRP Review Panel on Research Projects (RP2) comprising of external reviewers and WHO scientific staff reviewed and approved the scientific and technical content of the study (protocol ID, A65878). Ethics approval was obtained from the WHO Research Ethics Review Committee (ERC) and ethics review authorities of all participating sites (Federal Capital Territory Health Research Ethics Committee and Ondo State Ministry of Health Research Ethics Review Committee in Nigeria, and Makerere School of Health Sciences Research and Ethics Committee in Uganda.

### Study timeline

The time frame for the formative research and the development of prototypes for both SELMA and Passport to Safer Birth is two years, out of which approximately four months will be dedicated to the finalisation of research and ethical reviews at the WHO and at local institutional levels. The data collection and analyses are expected to be completed over a period of eight months. As the design of SELMA and Passport to Safer Birth will depend on the results from this phase (qualitative and service design research), the development of their prototypes will overlap with the final phase of data analyses. Report writing and result dissemination will occur after the formative phase is complete.

## Discussion

### Expected study outcomes

The main outcomes of this formative research will include: 1) an in-depth knowledge of care providers and health system issues that should be accounted for in the final design of SELMA; 2) identification of key moments where women’s experience of facility-based intrapartum care is shaped (“touch points”), and therefore where the desired connection to the facility could be established; and 3) improved understanding of expectations and preferences as well as motivators and incentives for behavioural change among health care providers and recipients, which will contribute to the development of the Passport to Safer Birth.

### How individuals (women and men) are affected by the public health need that the study will address

Over 99% of maternal and infant deaths and morbidities related to childbirth are recorded in low resource settings [34]. Most of these adverse outcomes could be mitigated by improving the coverage of effective intrapartum interventions to vulnerable populations. Yet, few innovative ways exist to improve access to quality intrapartum care in health facilities by women and their partners, particularly those who make effort to access skilled care. The views of women, and those of their partners, have been poorly acknowledged in care provision in most health facilities in low- and middle-income countries. There is increasing evidence to indicate that women value supportive birth companionship, privacy, familiar practices and respectful maternity care which are often not the norms in many health facilities. The BOLD project will be using innovative tools to promote access to effective care in a way that upholds the rights and dignity women and their partners during childbirth.

### Contribution of the study to identifying and/or reducing gender inequities in sexual and reproductive health care

Access to health care is limited for both poor men and women throughout the African continent, but women suffer the consequences of this limited access far more gravely, particularly during pregnancy and childbirth. The proposed research will explore opportunities to improve demand for quality intrapartum care by identifying key moments in the facility where women’s experience of care is shaped. The process of arriving at the ‘negotiated standards of care’ which underpins the development of Passport to Safer Birth will allow women to express their own priorities in labour and childbirth care, and encourage health managers and providers to respect such priorities. Application of the study findings will facilitate the development of an innovative set of service prototypes and/or tools towards promoting quality health care practices that will meet women’s reproductive health needs, and thus reduce inequities between men and women in sexual health and health care access.

### Measures to ensure inclusive community involvement

This formative research will involve strong participation of all eligible women, regardless of ethnicity or social status, within the community to achieve the set objectives. Therefore, all community communication and education strategies will be employed to ensure that women within all social strata are invited to participate. These strategies will include the use of posters and leaflets to inform the entire community about the research, interaction with community/opinion leaders, information dissemination at the selected health facilities, and when possible research staff will give talks at community forums and meetings.

### Main problems anticipated and proposed solutions

It is possible that healthcare providers (nurses, midwives, and doctors) may not feel comfortable discussing the quality of intrapartum care in their work place with their co-workers. However, interviews will be conducted in a private setting and the study team will remind participants that their names will not be linked to any responses and encourage the study participants to uphold confidentiality among their peers. Furthermore, the primary aim of the focus group discussions with providers is to explore the norms related to the provision of intrapartum monitoring and care in their facility. As such, the moderator of the focus group discussion will be trained to create a cordial and trusting environment that encourages the participants to willingly engage in the discussion. It is possible that identifying women in the facility-catchment areas, particularly in the urban setting, may be challenging. The study team will rely on the in-country partners and facility staff in both Nigeria and Uganda to identify the relevant facility-catchment areas from which to identify potential participants. It is also possible that women may not feel comfortable discussing childbirth in the FGDs and IDIs as childbirth may be considered a private matter. The study team will attempt to mitigate this concern by ensuring the data collectors for both the FGDs and IDIs are female.

### Applicability of results

The results of this study will inform the development of tools that will be relevant for women giving birth in settings similar to the research sites, which would include a large proportion of births in low-income countries. In addition, they will fill an urgent need for better information for women, clinicians and policy-makers in low resource setting with regard to innovative ways to improve quality of intrapartum care. As the formative research for SELMA will be conducted in the same facilities as the cohort study to inform the SELMA algorithm and tool, the findings from the formative research will be directly applicable to the development of SELMA. The SELMA development team will incorporate findings from the formative research directly into the SELMA labour algorithm at its final phase of development. For example, the final SELMA algorithm may include prompts or alerts related to specific barriers identified in the provision of intrapartum care by the formative research, such as the a reminder to conduct a specific type of physical examination or test, reminders to communicate with the woman and her family, or to consult with a colleague or higher ranking clinician. In effect, SELMA will be a more effective and adapted labour monitoring-to-action tool that provides clinicians with pertinent information relative to patient care.

Furthermore, the findings from the formative research will inform the development and design of the Passport to Safer Birth concept. As outlined in this protocol, the innovative Passport to Safer Birth concept will be developed in conjunction with the community and health care providers, and aims to create demand for quality and respectful care during facility-based labour and childbirth. An important output of this community-health system collaboration is the development of negotiated standards of intrapartum care which will be informed by the findings of this formative research (Table 1). The tools developed from this project (SELMA and Passport to Safer Birth will be integrated into a larger quality improvement approach (the BOLD strategy), which will then be tested in an intervention research study during the second phase of the project.

### Links with other projects

The proposed study is integral part of the BOLD project, a larger initiative with the overall goal of reducing adverse maternal and infant outcomes resulting from labour complications through research, design, and implementation of innovative tools. The BOLD project also includes the development of the SELMA algorithm and tool. In the future, the findings of this project will be used to guide the formulation of WHO guidelines on intrapartum care.

### Plans for dissemination of study findings

The results arising from the study will be published in a reputable, open access peer-reviewed journal. All publications will follow relevant external guidance such as the ‘Uniform Requirements for Submission of Manuscript to Biomedical Journals’ issued by the International Committee of Medical Journal Editors (ICMJE). Dissemination of results to participating institutions and communities will take place through meetings of stakeholders within the facilities and the communities. The results of the study will first be reported to collaborating investigators. Collaborating investigators will then disseminate local and collective results to their department and relevant authorities within the countries.

The project has a public website (www.boldinnovation.org) through which progress, activities and findings of the project will be documented and shared. Additionally, a detailed communication plan will be developed at the outset of the research activities.
